# Late-Onset Angioedema With Olanzapine in a Tertiary Hospital

**DOI:** 10.7759/cureus.65478

**Published:** 2024-07-26

**Authors:** Rishitha Kotla, Swapnil Aloney, Surabhi Borkar

**Affiliations:** 1 Psychiatry, Jawaharlal Nehru Medical College, Datta Meghe Institute of Higher Education and Research, Wardha, IND; 2 Psychiatry, Topiwala National Medical College (TNMC) and Bai Yamunabai Laxman Nair Charitable Hospital, Mumbai, IND

**Keywords:** aripiprazole, angioedema, late-onset, olanzapine, schizophrenia

## Abstract

A medical condition known as angioedema is characterized by sudden swelling of the mucosa, subcutaneous tissue, dermis, and submucosal tissues. If airway obstruction results in respiratory distress, this condition may be fatal. Histamine, bradykinin, and leukotrienes are just a few of the complex chemotactic mediators that play a role in the pathophysiology of angioedema and can lead to fluid buildup in deeper skin layers. Many things, such as medication side effects, genetic disorders, and allergic reactions, can cause angioedema. Olanzapine, an atypical antipsychotic mainly used to treat a few mental disorders, is one notable drug linked to angioedema. Angioedema is a documented side effect of olanzapine, albeit rare. Although the exact mechanism by which olanzapine causes angioedema is unknown, immunological-mediated or idiosyncratic reactions are thought to be involved. This study aims to review the current literature on the association between olanzapine and angioedema, including potential mechanisms of action and implications for clinical management. The possible risk factors, presentation, diagnosis, and treatment options for olanzapine-induced angioedema will also be discussed.

## Introduction

Angioedema is an anaphylactic skin condition associated with rapid swelling beneath dermal, subcutaneous, mucosal, and submucosal tissues [1]. At times, it also involves the respiratory and gastrointestinal systems, which can also be life-threatening if left unnoticed [2]. Nonsteroidal anti-inflammatory drugs (NSAIDs), angiotensin-converting enzyme inhibitors (ACEIs), angiotensin II antagonists, radiographic dyes, and opiates are notoriously known for causing these skin conditions. Several types of angioedema have been observed (acute allergic, nonallergic, idiopathic, hereditary, acquired C1 inhibitor deficiency, and vibratory angioedema) based on the type of exposure [3]. However, very rarely, antipsychotics (clozapine, olanzapine, iloperidone, haloperidol, quetiapine, paliperidone, ziprasidone, risperidone, and chlorpromazine) also tend to give the same transient effects like facial swelling, skin reactions, and other systemic effects [4].

Late-onset angioedema is a rare clinical condition that occurs for months or years and is more likely to be reported with ACEIs. Here, we report about a patient who developed late-onset angioedema after using olanzapine for three years. This was resolved by decreasing the dosage and cross-tapering it with another antipsychotic from the same class. Understanding this association is important for healthcare professionals involved in the care of patients taking olanzapine to promptly recognize and manage this potentially life-threatening adverse event. The aim of this case report is to demonstrate the rare finding of the onset of angioedema post-chronic use of olanzapine in a patient who is maintaining well.

## Case presentation

A 25-year-old female Bachelor of Science (BSc) student came to the hospital three years ago, complaining of being fearful about her surroundings and suspicious that people around her were talking about her. She also reported her sleep disturbances and occasional irritability without provocation, along with the auditory hallucinations being distressful enough to make her aggressive toward the family members for more than a month, and that she needs medication for them. Her symptoms were assessed, and she was diagnosed with schizophrenia according to the International Statistical Classification of Diseases and Related Health Problems (ICD-11). Her father was also known to have a psychiatric illness (bipolar affective disorder) and was on regular medication. She had no history of any head trauma, alcohol, or substance use.

On a mental status examination, she was alert, suspicious, fully oriented, and cooperative. Her mood was irritable. Speech pace and quality were average, with a delusion of reference and persecution. She had no insight into her condition. Her general physical and neurological examination was quite unremarkable. All routine investigations, including the electrocardiogram and chest X-ray, were done, which revealed that they were within normal limits. She was started on olanzapine at lower doses, which was titrated to the desired dose (from 5 mg to 10 mg). She reported having second-person auditory hallucinations that were audible and familiar. These were the voices of her two classmates passing derogatory comments to her. Despite the mild weight gain and occasional sedation on medication, she stated that she has observed substantial improvement in her psychotic symptoms since she was in regular compliance with it. She was maintaining well on medication until a month ago, when she came with complaints of periorbital swelling, lipedema, and rashes all over her skin. There were no significant dietary changes, lifestyle changes, or new medications, as assured by the mother.

**Figure 1:**
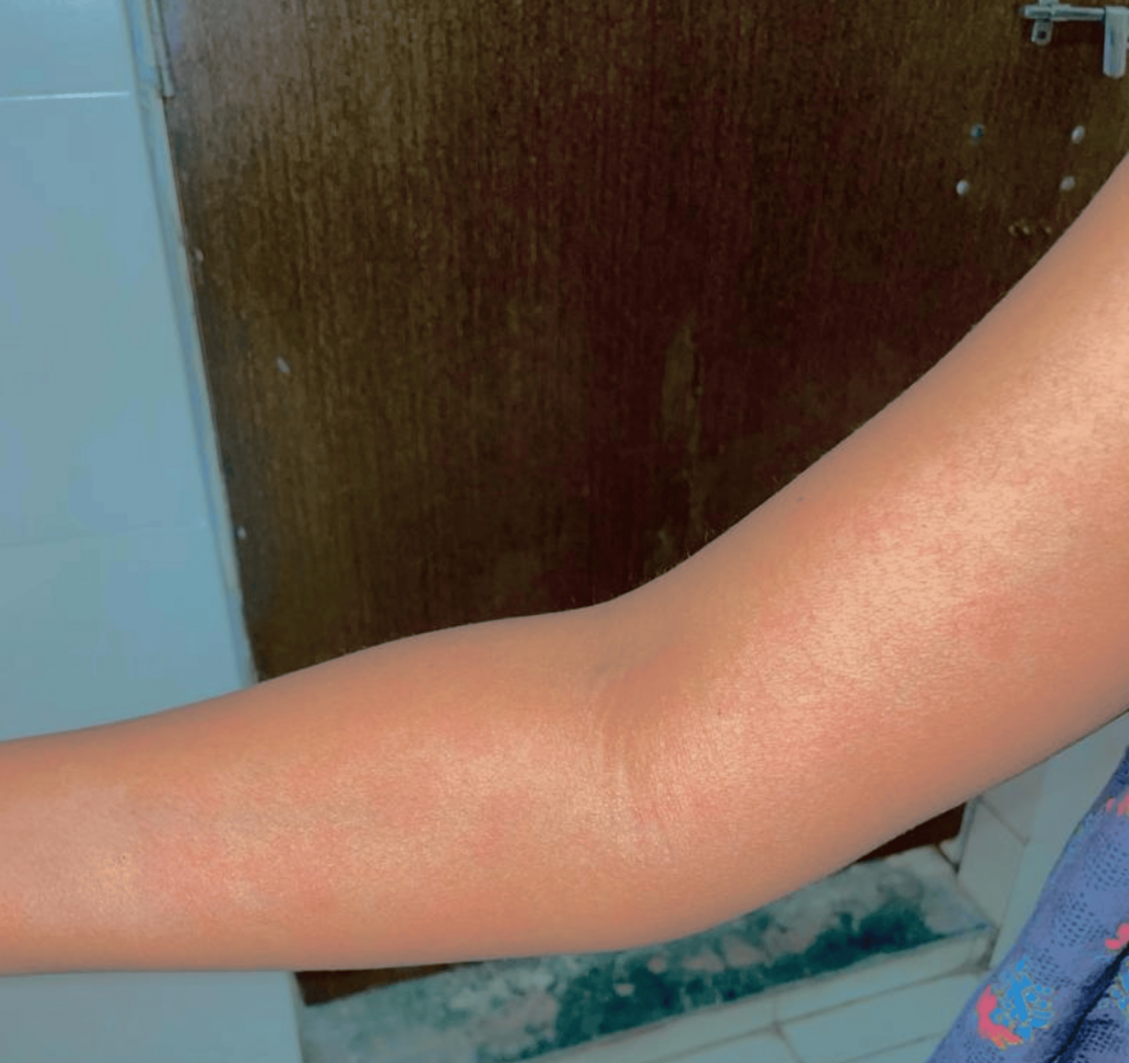
Olanzapine-induced angioedema

**Figure 2:**
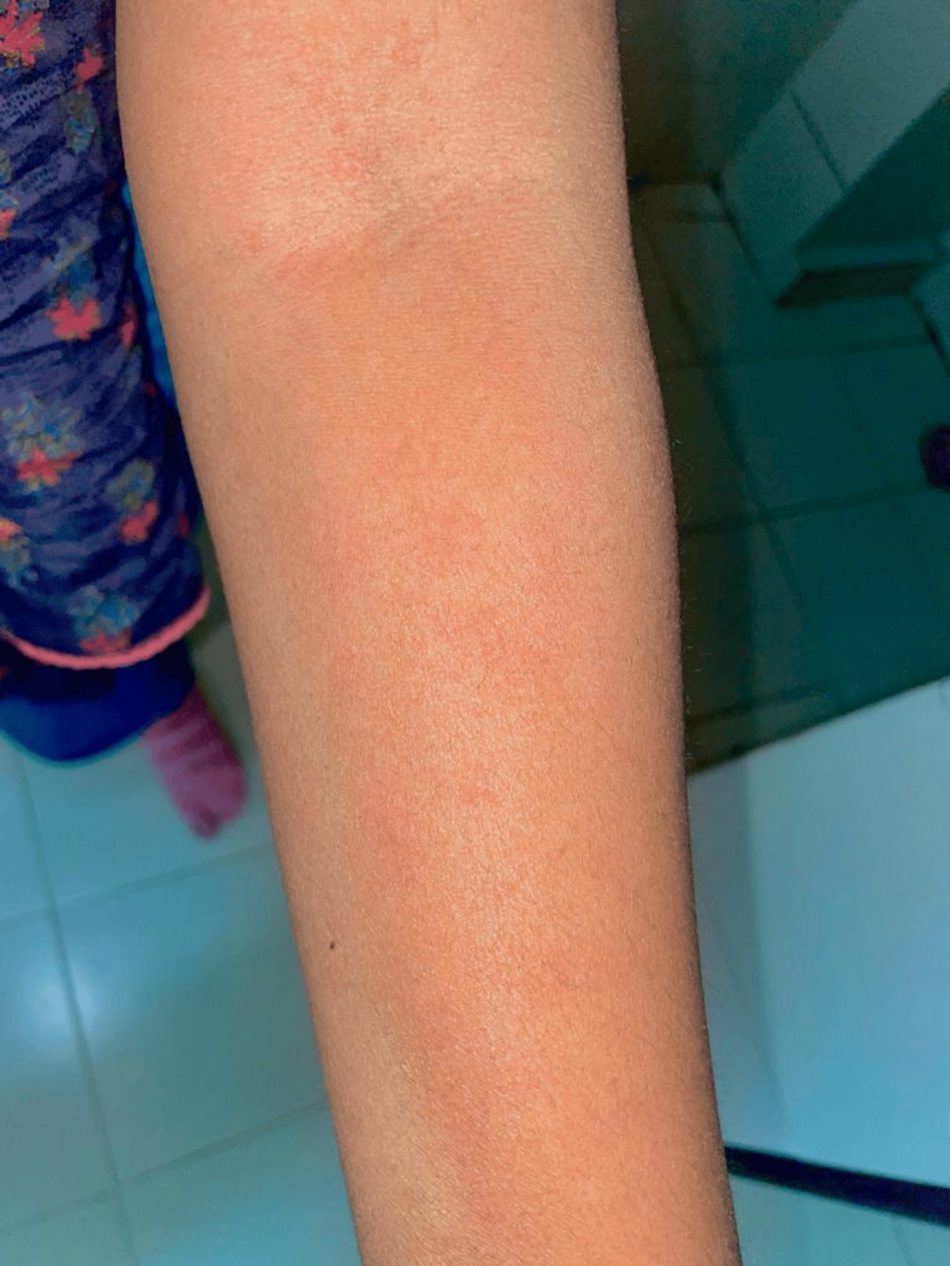
Sudden onset of skin rashes with hives after long-term olanzapine use

Visually, the hives, along with the above symptoms, gave a vague presentation of angioedema, and to rule out other causes like drug reactions with eosinophilia and systemic symptoms (DRESS) syndrome, blood investigations were advised, which were within normal limits. A dermatological opinion was taken to rule out other differential diagnoses where skin biopsy was advised but wasn’t done as she was not cooperative for it. She was given a tapering dose of prednisolone and antihistaminics, along with a cross-tapering regime of olanzapine with aripiprazole. Within five days, she had a complete resolution of her cutaneous symptoms without any relapse of her psychotic symptoms. Figure 1 and Figure 2 show olanzapine-induced angioedema.

## Discussion

Angioedema is a transient edema that is non-pitting in nature and affects the face, lips, extremities, and, at times, the oral cavity, including the larynx, followed by respiratory and gut symptoms [5]. It is a type of hypersensitivity reaction (anaphylactic) that tends to happen on exposure to an allergen. The confounding factors could be anything from hereditary to acquired, allergic, or drug-induced. The onset of drug-induced angioedema is more often seen in the early introduction of a drug. But, in our case, olanzapine was used for three years with minimal side effects. It is inferred that the drug triggered an IgE-mediated mast cell degranulation, causing the release of inflammatory mediators such as histamine, serotonin, and bradykinins, which increase vascular permeability and cause angioedema [6]. Olanzapine is commonly used for schizophrenia, mania, and a few other behavioral symptoms. In this case, swelling and skin rashes correlated with the use of olanzapine tablets and how their discontinuation relieved them. There are notably few cases reported with such a picture, as they are infrequently reported adverse reactions [7]. Multiple articles have described fever and hepatitis, eruptive xanthomas, and nonpruritic purpura when on olanzapine and the disappearance of lesions on discontinuation [8-9].

There have been multiple reports about clozapine and other antipsychotic-induced angioedema, but not many were related to olanzapine monotherapy [10]. A possible explanation for this might be an unidentified new vulnerability factor that was going on at the time of the development of angioedema and evidently persisted during the introduction of olanzapine, which may have subsided by the time aripiprazole was started. Angioedema can occur even many years after uneventful drug use [11]. However, there has been no evident mechanism of action to prove why this condition emerges, but it is also known that type I hypersensitivity reaction can begin at any time in life and are often overlooked [12]. Clinicians should be aware of such skin conditions that are caused by olanzapine and manage it timely so that there is no irregularity with compliance to antipsychotics as it also has propensity for causing serious health conditions like DRESS syndrome if neglected [13].

## Conclusions

Angioedema due to any causative factor can be a swelling underlying the skin, which in this case has been induced by the use of an antipsychotic like olanzapine after a long duration. The mechanism of action, however, is evidently due to interference with different chemotactic mediators, each requiring management based on severity. Its treatment includes antihistamines, corticosteroids, and rarely epinephrine for severe allergic reactions. It is important for a healthcare professional to be accurate in their diagnosis, side effect monitoring, and early management.
